# Increased Expression of Thymic Stromal Lymphopoietin in Chronic Constriction Injury of Rat Nerve

**DOI:** 10.3390/ijms22137105

**Published:** 2021-07-01

**Authors:** Chieh-Hsin Wu, Chun-Ching Lu, Chao-Lan Huang, Ming-Kung Wu, Ying-Yi Lu

**Affiliations:** 1 Division of Neurosurgery, Department of Surgery, Kaohsiung Medical University Hospital, Kaohsiung 807, Taiwan; wujoeys@gmail.com; 2 Department of Surgery, School of Medicine, College of Medicine, Kaohsiung Medical University, Kaohsiung 807, Taiwan; 3 Department of Orthopaedics and Traumatology, Taipei Veterans General Hospital, Taipei 112, Taiwan; ckluking@gmail.com; 4 Department of Orthopaedics, School of Medicine, National Yang Ming Chiao Tung University, Taipei 112, Taiwan; 5 Department of Anesthesiology, Taipei Veterans General Hospital, Taipei 112, Taiwan; clhuang11@vghtpe.gov.tw; 6 Department of Anesthesiology, School of Medicine, National Yang Ming Chiao Tung University, Taipei 112, Taiwan; 7 Department of Psychiatry, Kaohsiung Chang Gung Memorial Hospital and Chang Gung University College of Medicine, Kaohsiung 833, Taiwan; mingkung@cgmh.org.tw; 8 Department of Dermatology, Kaohsiung Veterans General Hospital, Kaohsiung 813, Taiwan; 9 Graduate Institute of Medicine, College of Medicine, Kaohsiung Medical University, Kaohsiung 807, Taiwan; 10 Department of Health and Beauty, Shu-Zen Junior College of Medicine and Management, Kaohsiung 821, Taiwan

**Keywords:** thymic stromal lymphopoietin (TSLP), dorsal root ganglions (DRG), neuropathic pain, chronic constriction injury (CCI)

## Abstract

Thymic stromal lymphopoietin (TSLP) is a well-known cytokine for T helper 2 inflammatory responses. A nerve injury activates the neuroinflammation cascade and neuron–glia interaction in dorsal root ganglions (DRG)s, leading to neuropathic pain. Therefore, this study was to investigate the role of TSLP after nerve injury. Male Sprague-Dawley rats were divided as an experimental group with chronic constriction injury (CCI) to the sciatic nerve and a control group. The mechanical pain threshold response was determined by calibration forceps. After assessment of mechanical allodynia, the ipsilateral spinal cord, DRG, sciatic nerve and skin were harvested. Immunofluorescence staining was performed to identify cell types with various markers. Western blot analyses were performed to evaluate protein expressions. Mechanical allodynia developed after CCI and persisted for the next 14 days. Astrocyte reactions occurred and continued until day 14, too. After CCI, DRG and the sciatic nerve also had significantly increased expressions of TSLP/TSLP-R/STAT5. The TSLPR was localized to sensory neuronal endings innervating the skin. This study is the first to demonstrate that the TSLP complex and the STAT5 pathway in nerve are potential therapeutic targets because of their roles in pain regulation after nerve injury.

## 1. Introduction

Sciatica, a common medical complaint, is characterized by radicular leg pain radiating along the sciatic distribution [1,2,3]. Arising from nerve injury in somatosensory system lesions, sciatica is an exemplary cause of neuropathic pain [4,5,6] which may compromise quality of life to an extreme degree. Neuropathic pain develops not only in the area supplied with the injured nerve, but also in near areas innervated with other intact nerves; nerve sprouting can induce persistent tactile evoked allodynia [7,8]. Dorsal root ganglion (DRG) neurons can connect transmission between peripheral and central sensitization, which serve as a bridge between stimulus and spinal cord. Primary neurons in DRG may release abnormal activity, enhance peripheral sensitization and affect expression of nociceptors, ion channels or enzymes, which is associated with pain induced by nerve injury [9]. Neuroinflammation plays vital roles in the course of chronic sciatica; inflammatory factors induce excess glial cells activation to cause neuropathic pain in damaged nerves [10,11,12]. Previous studies also indicate that intervertebral disc cells lead mononuclear cells or macrophages to invade into intervertebral disc nerves, and then induce inflammatory cell oversecretion [13,14,15,16].

Thymic stromal lymphopoietin (TSLP), a well-known cytokine and proinflammatory factor, is important for the generation and maintenance of T helper 2 (Th2) inflammatory responses through a ternary complex comprising TSLP, the TSLP receptor (TSLPR) and interleukin-7 receptor (IL-7R) α chain [17]. In patients with middle cerebral artery occlusion, elevated TSLP expression is identified in neurons and glial cells [18]. Although a few reports have discussed the relevance of TSLP to the nervous system, none have discussed its relevance to neuropathic pain. Only the TSLP-positive rate has been reported as positively associated with Visual Analog Scale (VAS) pain score in patients with lumbar disc degeneration [19].

Currently, the complex mechanisms in neuropathic pain remain largely unknown; specifically, no studies have investigated whether TSLP is associated with neuropathic pain. Therefore, this study aimed to investigate the role of TSLP in neuropathic pain though chronic constriction injury (CCI) of sciatic nerve in a rat model.

## 2. Results

### 2.1. Expression of TSLP in DRG Was Increased After CCI

**Figure 1:**
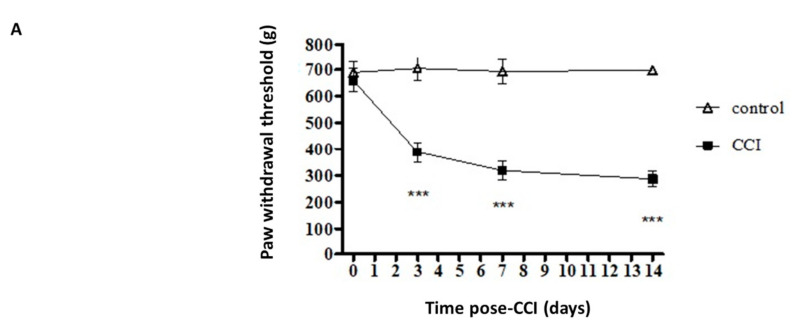

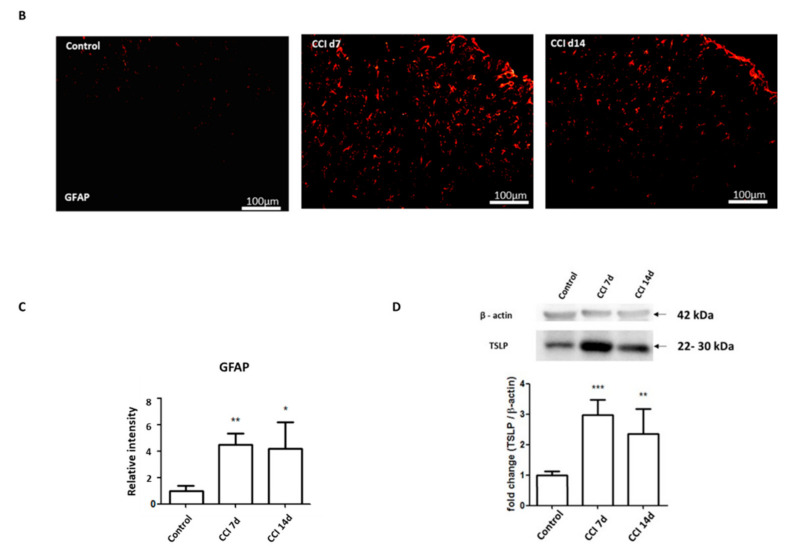
CCI induced mechanical allodynia and increased TSLP levels in L4/5 DRGs. (**A**) Behavioral data demonstrating the reduction of PWT though calibrated forceps test after CCI. The control group received no sciatic nerve ligation. N = 12 for each group. *** *p* < 0.001 vs. control group analyzed by ANOVA test. (**B**) Immunofluorescence staining showing increased astrocyte reaction in the L4/5 dorsal spinal cord of rats after CCI. Scale bar, 100 μm. (**C**) Relative fluorescence intensity of GFAP. (**D**) Western blot analysis showing increased TSLP protein expression in L4/5 DRGs of rats after CCI. Each band density was quantitated, normalized with its own β-actin. Values are presented as mean ± SEM. N = 6 for each group. * *p* < 0.05, ** *p* < 0.01 and *** *p* < 0.001 vs. control group analyzed by ANOVA test.

After the rat model of CCI was established, calibrated forceps testing was applied to measure the mechanical threshold for pain. The control group received no sciatic nerve ligation. Paw withdrawal (pain) thresholds (PWT) in the control group did not vary from baseline (d0). In the experimental group, CCI resulted in a large decrease in the PWT at 3 d post CCI, and the decrease last for 14 d post CCI (Figure 1A). Astrocyte reaction (upregulation and astrogliosis of glial fibrillary acidic protein (GFAP)) has also been observed in various injury conditions associated with enhanced pain states. Figure 1B shows the results of GFAP staining performed to detect reactive astrocytes. According to the staining results, CCI induced significant increases in astrocyte reaction at 7 d and 14 d post CCI (Figure 1C). Western blot analysis performed in triplicate also showed significant increases in protein expressions of TSLP in the ipsilateral DRG after CCI; the increases were detectable at 7 d and persisted until 14 d post CCI (Figure 1D).

### 2.2. Expression of TSLPR in Damaged Neurons with TSLP After CCI

**Figure 2:**
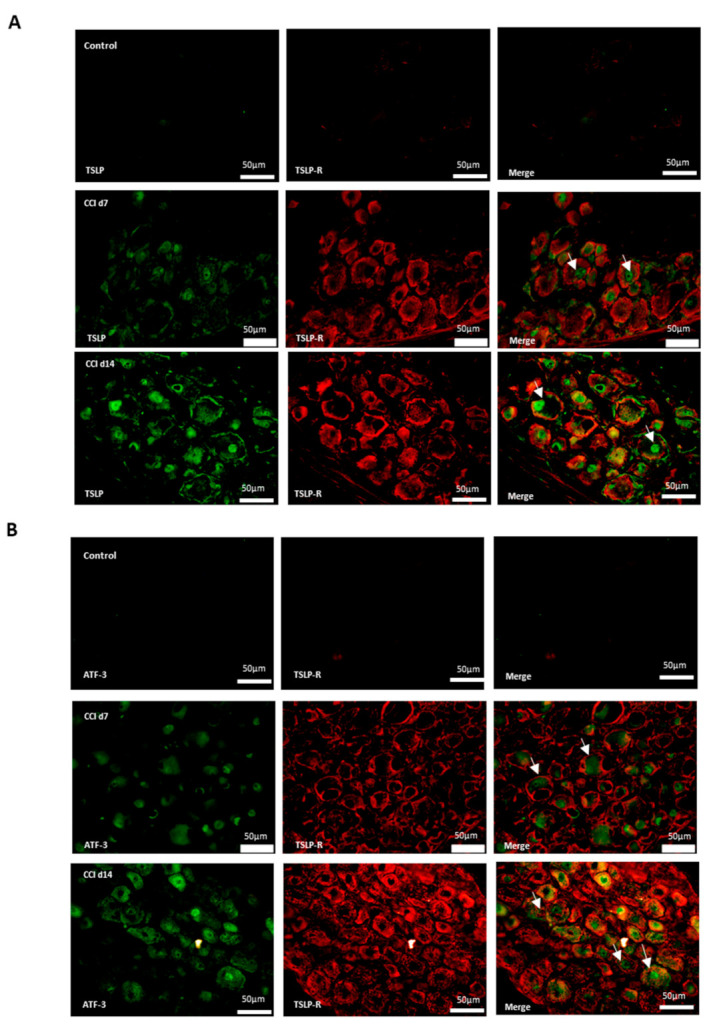
TSLP receptor expressed in injured DRG neurons. Double-labeled immunofluorescence imaging was performed in L4/5 DRGs of rats at 7 days and 14 days after CCI. (**A**) Representative double immunofluorescence labelling for TSLP (green) with TSLPR (red). Scale bar, 50 μm. (**B**) Representative double immunofluorescence labelling results for TSLPR (red) with ATF-3 (green). Scale bar, 50 μm. Pairs of images are merged. White arrows indicate double-labeled cells. N = 6 per group.

By inducing signaling changes in the sciatic nerve, DRG, spinal cord dorsal horn and brain, a sciatic nerve injury can influence both the central and peripheral nervous systems. Signaling of TSLP requires a distinctive TSLPR, a heterodimer consisting of IL-7Rα and a common γ receptor-like chain [17]; TSLP expression is positively proportional to TSLPR expression [20,21]. Therefore, immunofluorescence staining was performed to label TSLPR. Figure 2A shows that TSLPR was co-expressed with TSLP. Activated transcription factor-3 (ATF-3), a marker of injured or hyperactivated peripheral neurons, was co-localized with TSLPR and increased compared with control group (Figure 2B), which suggested that TSLPR was expressed in damaged DRG neurons following nerve injury [22].

### 2.3. Expression of TSLP in DRGs with Neuron–Glia Interaction After CCI

**Figure 3:**
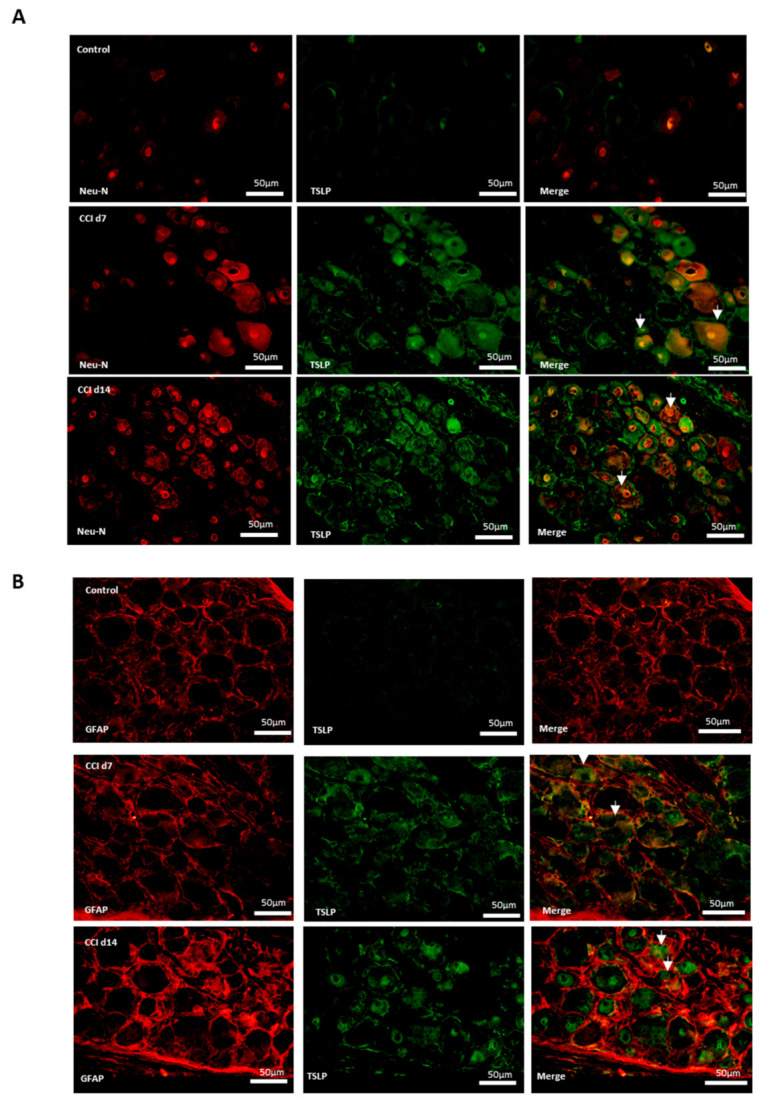

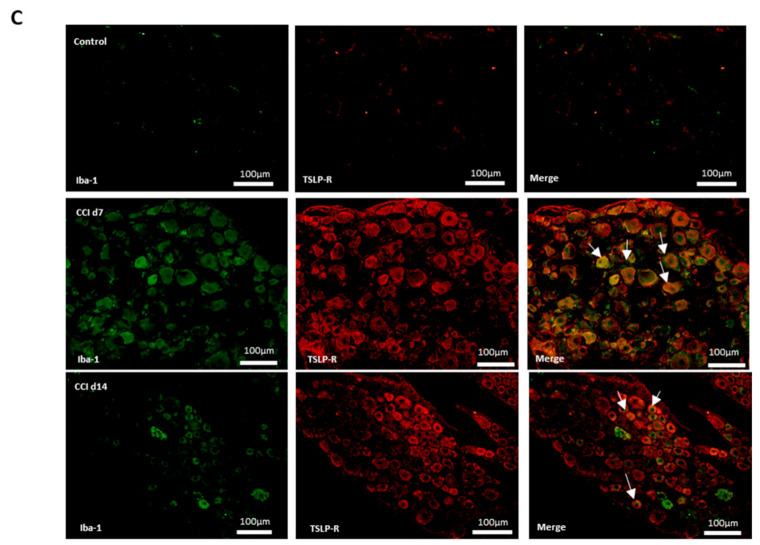
TSLP expressed in DRGs with neuron–glia interaction after CCI. Double-labeled immunofluorescence imaging was performed in L4/5 DRGs of rats at 7 days and 14 days after CCI. (**A**) Representative double-labeled immunofluorescence images showing TSLP (green) with neuron marker NeuN (red). Scale bar, 50 μm. (**B**) Representative double-labeled immunofluorescence images showing TSLP (green) with satellite glial-cell marker GFAP (red). Scale bar, 50 μm. (**C**) Representative double immunofluorescence labelling results for TSLPR (red) with iba1 (green). Scale bar, 100 μm. Pairs of images are merged. White arrows indicate doubled-labeled cells. N = 6 per group.

Additionally, neurons, satellite-glia cells and macrophages were labeled NeuN, GFAP and iba1, respectively. Figure 3 shows that there were neither TSLP-expressing neurons (Figure 3A), satellite-glia cells (Figure 3B), nor TSLPR-expressing macrophages (Figure 3C) in the control group. After CCI, TSLP increased mainly in the cytoplasm and cell membrane of neurons and satellite-glia cells when TSLPR increased on (activated) macrophages, which suggested that TSLP expression had functional roles in neuron–glia cell interaction in DRGs.

### 2.4. Expression of TSLPR in Sensory Terminals in Ipsilateral Skin After CCI

**Figure 4:**
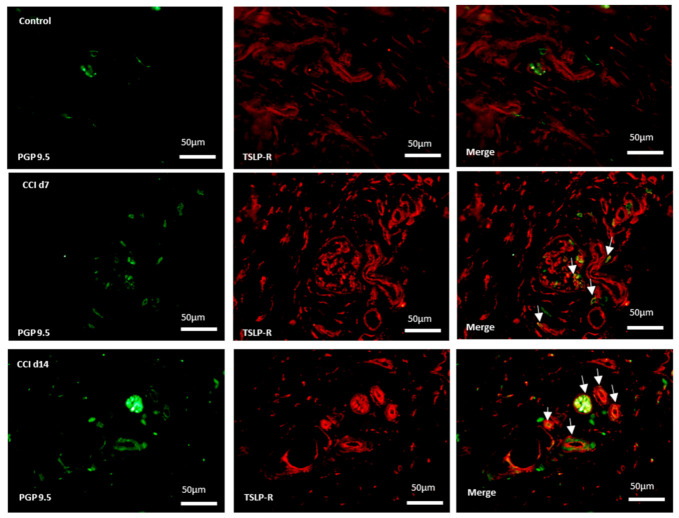
Expression of TSLPR in sensory terminals in ipsilateral skin after CCI. Representative double immunofluorescence labelling results for TSLPR (red) and PGP 9.5 (green) in the hind-paw skin. Scale bar, 50 μm. Pairs of images are merged. White arrows indicate doubled-labeled cells. N = 6 per group.

Skin nociceptors supplied by adjacent intact nerves become sensitized to mechanical stimulus after nerve injury [23]. Sympathetic fibers can reportedly invade the upper dermis of the skin, where they interact with sensory fibers. If TSLP/TSLPR complex mediate sensory signal, they might confine to primary afferent nerve endings in the skin. We further performed immunofluorescence staining for TSLPR and PGP9.5 (pan-neuronal fiber marker) on the ipsilateral skin in CCI-rats. The TSLPR-expressing neurons increased in the ipsilateral skin after CCI (Figure 4), which suggests that TSLPRs were expressed in a subset of sensory neurons innervating the skin, possible mediating pain transduction.

### 2.5. Signaling of STAT5 Involved in DRG Neurons and the Sciatic Nerve After CCI

**Figure 5:**
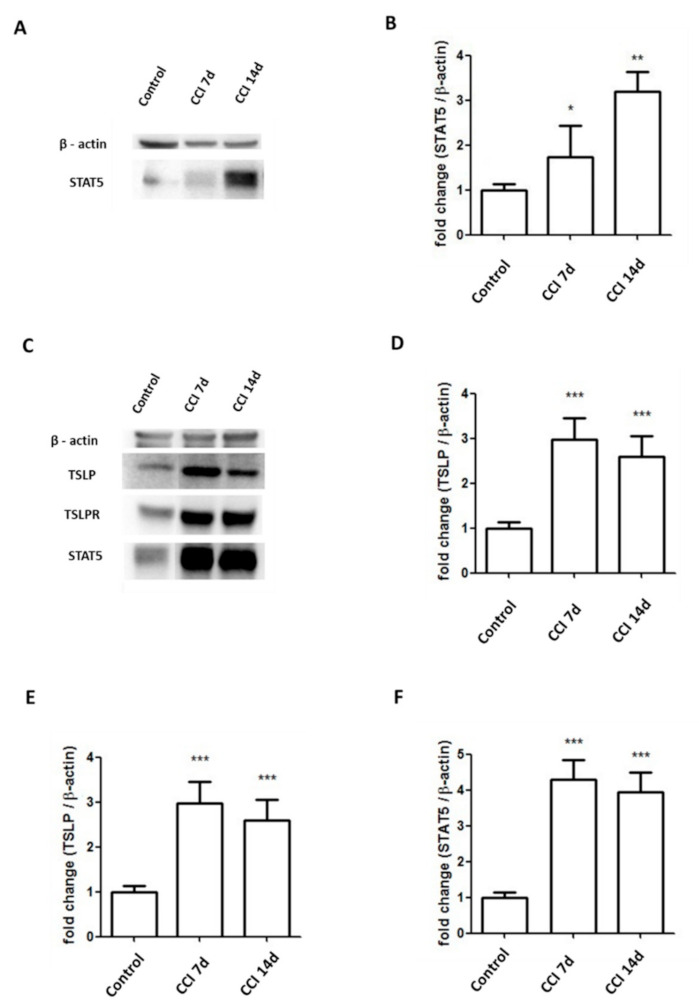
Expressions of STAT5 increased in sciatic nerve and DRGs in CCI rats. (**A**,**B**) Levels of STAT5 in the L4/5 DRGs of rats increased at 7 days and 14 days after CCI. Representative Western blot results. Each band density was quantitated, normalized with its own β-actin. (**C**–**F**) Levels of TSLP, TSLPR, STAT5 in the sciatic nerve of rats increased at 7 days and 14 days after CCI. Representative Western blot results. Each band density was quantitated, normalized with its own β-actin. Values are presented as means ± SEM. N = 6 for each group. * *p* < 0.05, ** *p* < 0.01 and *** *p* < 0.001 vs. control group analyzed by ANOVA test.

Through binding with TSLPR, TSLP can activate STAT5 to induce an inflammatory response [24,25,26]. Compared to the control group, the CCI group had significance at nearly 3-fold higher expressions of STAT5 in ipsilateral DRGs (Figure 5A,B). Additionally, the CCI group had significance at nearly 3-fold, 3.5-fold, 4-fold greater expressions of TSLP, TSLPR and STAT5, respectively, in the sciatic nerve after CCI (Figure 5C–F).

## 3. Discussion

A CCI in the sciatic nerve causes persistent allodynia or hyperalgesia similar to those in patients with neuropathic pain [27]. In the present study, CCI to the sciatic nerve significantly reduced the pain thresholds of rats, and the reduced pain threshold persisted until 14 d post CCI at least, which is in line with earlier reports [28]. Additionally, CCI induced increased TSLP expression in L4/L5 DRGs where peripheral sensory neurons are located. Expressions of downstream STAT5 increased in DRGs and sciatic nerves. TSLPRs were confined to sensory neuronal terminals innervating the skin. Our findings suggest that TSLP may contribute to regulate pain signals.

Although neuropathic pain has been studied intensively, the exact underlying mechanism remains unclear, and no effective drug for mitigating neuropathic pain is currently available [5]. In the CCI rats, mechanical allodynia developed at 3 days after injury, and the allodynia continued until 14 days after injury. Expression of TSLP protein in DRGs also increased and persisted for up to 14 days after CCI (coinciding with mechanical sensitivity). The TSLP was localized with TSLPR, which was observed in ATF3-immunoreactive DRG neurons. Therefore, we hypothesize that DRGs produce TSLP protein in response to nerve injury.

Both central and peripheral sensitization are pathological contributors to neuropathic pain [6,29,30]. Through release of neurotransmitters in the primary nociceptor afferent at the central terminal, the central nervous system is affected by retrograde sensitization from damaged peripheral sensory neurons [31,32]. Glial activation is another well-known contributor to the development and maintenance of neuropathic pain [33]. In particular, nerve injury is a known cause of microglial activation resulting in cytokine or chemokine production that increases the excitability of dorsal horn projection neurons [34]. Astrocyte activation may be responsible for long-term maintenance of chronic pain [35]. The overall signaling cascade spontaneously activate to modulate pain by sensitizing the dorsal horn. Activity-dependent plasticity in spinal cord neurons induces central sensitization, thus leading to pathological hyperalgesia. Our model revealed increased expression of GFAP in the spinal cord (coinciding with mechanical sensitivity and expression of TSLP), which is consistent with previous reports [29].

Increased excitability of DRG neurons is a major mechanism of neuropathic pain, enhancing nociceptive inputs to the supraspinal sites [36]. Sensory neurons are encompassed by satellite glial cells (SGC) and other non-neuronal cells in DRGs. Peripheral nerve injury can stimulate the release of inflammatory factors from neurons, conceivably alter neuronal excitability [37], and then enhance communication between neurons and SGCs in a persistent pain condition [38]. Therefore, injury initiates SGCs proliferation or activation and neuronal death in DRGs [39,40,41,42]. In parallel with activation of dorsal horn microglia, residual macrophages in DRGs rapidly proliferate, activate, increase their organelle content, become hypertrophic, and release mediators to sensitize neurons after injury, suggesting a neuron–glia interaction [43,44]. The macrophages in DRG are required for initiation and maintenance of mechanical allodynia after nerve injury [45]. Moreover, active macrophages act as neurosupportive cells by providing neuroprotection and by scavenging cellular debris resulting from injury [46]. Compared to small-C neurons, large-A neurons are more likely to be influenced by macrophages [47]. Our study showed that TSLP was expressed on neurons and satellite glial cells, whereas TSLPR was expressed on activated macrophages. Therefore, the involvement of TSLP in neuron–glia interaction in DRGs through TSLPR may be an underlying mechanism to increase mechanical sensitivity.

Through the ternary complex (TSLP/TSLPR/IL-7Rα) and STAT5 activation, TSLP can trigger differentiation of inflammatory Th2 cells in rats [26]. In the CNS, choroid plexus epithelial cells and astrocytes can yield TSLP to regulate Th2 cells co-expressed with ternary complex in the perivascular and meningeal dendritic cell network [48]. In human, the ternary complex induces target genes expression by activating STAT3 or in a Janus kinase (JAK)-independent manner [26]. In our study, higher levels of TSLP and TSLPR in the DRGs and sciatic nerves of CCI group were observed compared with the control group. Additionally, the increases were accompanied by increased STAT5. Further, we showed that the TSLP/TSLPR was expressed in neuron–glia interaction. These data suggest that, in CCI, the TSLP may regulate neuroinflammatory processes through STAT5 pathway.

The limitation in our study is that the CCI model was the only involved neuropathic pain model. Different models of neuropathy could be performed in future studies to validate our results.

## 4. Materials and Methods

### 4.1. Animal Model

Purchased from the National Animal Center (Taiwan), male Sprague-Dawley rats (weight, 300–350 g) were housed in a room with a relative humidity of 70%, a temperature of 22 + 1 °C, and a 12-h light–dark cycle. Throughout the experiment, the rats were given ad libitum access to normal food and water. As described by Bennett and Xie, the CCI model of neuropathic pain was established (1988) [27]. In brief, the sciatic nerve was exposed at the mid-thigh level proximal to the sciatic trifurcation after rats were anaesthetized with Zolitil 50 (50 mg/kg) (Virbac, Carros, France; 06516) by intraperitoneal injection. Without compromising the vascular supply, four chromic gut ligatures (4/0) (ETHICONNew Jersey, USA; VE601) were loosely tied around the nerve at intervals of 1–2 mm. A CCI was induced in the twelve rats in the experimental group. The control group received sciatic nerve exposure without ligation (n = 12). The control group was run in parallel with experimental group. After the study, the rats were euthanized by injection of Zolitil 50 (100 mg/kg) and tissues were harvested. The protocol for use of the animals in this experiment was approved by the Institutional Animal Care and Use Committee of Kaohsiung Medical University; all procedures were performed based to ethical guidelines for the care and use of laboratory animals.

### 4.2. Calibrated Forceps Testing

The mechanical threshold response was evaluated with calibrated forceps (Bioseb, Vitrolles, France) and obtained from the same rats at different time points. Through this algometer, quantifiable mechanical stimulation was induced in a linear scale. The effect of the stimulation on each hind paw was measured three times using the mechanical threshold test described in Luis-Delgado et al. [49], which has been validated as sensitive and reliable. The maximum force utilized to the paw was defined as the grams (g) of force displayed on the dynamometer at the time of withdrawal. Withdrawal threshold was expressed as the mean ± SEM per group.

### 4.3. Immunofluorescence Staining

Tissue sections (thickness, 5 µm) of the 4th and 5th lumbar DRG, spinal dorsal horn and innervated skin were dried and then incubated in blocking buffer containing 0.2% Triton X-100 and 1.5% normal goat serum in PBS at room temperature. Theses sections were washed twice with PBS, then incubated with the primary antibodies (TSLP (1:100; Sigma; PRS4025), TSLPR (1:500; Sigma; WH0064109M3), GFAP (1:200; Sigma; G3893), NeuN (1:400; Millipore; MAB377), iba1 (1:100; proteintech; 10904-1-AP), ATF3 (1:500, Sigma; HPA001562), PGP9.5 (1:200; proteintech; 14730-1-AP)) at 4 °C overnight, further washed three times with PBS, and replaced in secondary antibodies at room temperature for 2 h. Negative controls in which the primary antibody was omitted were conducted. All immunofluorescence staining was obtained in a minimum of 4–6 serial sections for each rat. Theses sections were photographed by using a fluorescence microscope (Olympus, Commonwealth of Pennsylvania, USA, U-RFL-T). The fluorescence intensity of GFAP were captured from 5–6 randomly chosen fields in each section (the medial superficial dorsal horn laminae I–III) and measured with Image J (National institutes of Health, Bethesda, MA, USA). The background intensity was subtracted in each section and the intensity was presented as fold increase compared to the control.

### 4.4. Western Blot

The DRG and sciatic nerve specimens were homogenized in protein lysis buffer with protease inhibitors and then incubated at room temperature for 30 min. At 4 °C, those samples were centrifuged for 30 min at 13,000× *g* rpm. Protein concentration of supernatants was determined by the BCA Protein Assay Kit. Further, equal amounts of total proteins and 5× SDS sample buffer were mixed, boiled for 10 min, separated by 8–12% SDS-PAGE and then transferred to PVDF membranes. After blocking in TBST and 5% non-fat milk at room temperature for 1 h, the membranes were incubated overnight at 4 °C with various primary antibodies (TSLP (1:200; Sigma; PRS4025), TSLPR (1:500; Sigma; WH0064109M3), STAT5 (1:500; Abcam; ab230670), β-actin (1:10,000; Millipore; MAB1501R)). After six washes for 5 min each, an HRP-conjugated secondary antibody was used at room temperature for 1.5 h. Peroxidase activity was identified through the ECL Western Blotting Detection kit and the MiniChemi™ chemiluminescent imaging and analysis system (Sage Creation Science, Beijing, China).

### 4.5. Data Analyses

All outcome assessments were obtained by a trained technician who was blinded to the study group. All data in the experiment were expressed as mean ± SEM. Differences among groups were analyzed by one-way ANOVA following normal distribution, in which a *p*-value of less than 0.05 was considered statistically significant. All analyses were repeated at least three times, with representative results being shown. All of the above analyses were performed with SPSSstatistical software (V24.0, SPSS Inc., Chicago, IL, USA).

## 5. Conclusions

Our data demonstrated that sciatic nerve injury in rats lead to increased TSLP expression in DRG neurons. In primary sensory neurons, TSLP expression might have an association with mechanical allodynia observed in the experimental animals with possible neuron–glia interactions in DGRs. After nerve injury, expressions of TSLPR and STAT5 were increased in DRGs and in the sciatic nerve; the TSLPRs developed in sensory neuronal terminals innervating the skin. This observational study is the first to demonstrate evidence of the potential functional role of TSLP in regulating neuropathic pain.

## Data Availability

All data generated or analyzed during this study are included in this published article.
